# Correlation of immune cell subsets in the tumor microenvironment and peripheral blood with immunotherapy response in esophageal squamous cell carcinoma

**DOI:** 10.3389/fimmu.2025.1633748

**Published:** 2025-10-17

**Authors:** Wei Chen, Lian Gong, Yahu Li, Mengyao Wu, Min Tao

**Affiliations:** ^1^ Department of Oncology, Nantong Tumor Hospital, Tumor Hospital Affiliated to Nantong University, Nantong, China; ^2^ Department of Oncology, Taicang Loujiang New City Hospital (Ruijin Hospital, Taicang), Suzhou, China; ^3^ Department of Oncology, Longgang District Third People’s Hospital, Shenzhen, China; ^4^ Department of Oncology, The First Affiliated Hospital of Soochow University, Suzhou, China; ^5^ Department of Oncology, The Fourth Affiliated Hospital of Soochow University, Suzhou, China

**Keywords:** esophageal squamous cell carcinoma, tumor microenvironment, T cell immunoglobulin and mucin domain molecule 3, immunotherapy, flow cytometry

## Abstract

**Background:**

Esophageal squamous cell carcinoma (ESCC) is commonly diagnosed at an advanced stage, where conventional chemoradiotherapy offers only limited clinical benefit. Immune checkpoint inhibitors targeting the tumor microenvironment (TME) have demonstrated substantial therapeutic potential; however, reliable biomarkers for predicting therapeutic outcomes remain unclear.

**Methods:**

Single-cell RNA sequencing dataset for ESCC was obtained from the GEO database and analyzed using the Seurat R package to evaluate gene expression in tumor and adjacent tissues. Additionally, flow cytometry was used to assess immune cell subsets in peripheral blood samples from patients undergoing immunotherapy. Statistical analyses, including survival analysis and the Kruskal-Wallis test, were conducted to investigate the association between immune cell subsets and treatment efficacy.

**Results:**

In tumor tissues, immune subsets were significantly enriched compared with adjacent tissues, including CD8^+^ T cells with exhaustion (CD39, TIM3, PD-1) or activation/tissue residency (CD137, CD103) features; CD4^+^ T cells with activation (CD134, CD137) or regulatory (FOXP3) phenotypes; and dendritic cells expressing TIM3 or CD103. In peripheral blood, a median change in TIM3^+^ CD8^+^ T cells of 3.35% was observed following immunotherapy. Patients with changes exceeding this threshold experienced shorter progression-free survival (PFS) compared to those with lower changes (5.0 vs. 8.5 months, P = 0.024). Furthermore, TIM3^+^ CD8^+^ T cell changes were markedly reduced in patients achieving complete or partial responses compared to those with progressive disease.

**Conclusions:**

TIM3^+^ CD8^+^ T cells are a promising predictive biomarker for immunotherapy outcomes in ESCC. These findings highlight their potential to guide personalized treatment strategies in clinical practice.

## Introduction

Esophageal cancer (EC) is the eighth most common malignancy worldwide and ranks sixth in cancer-related mortality (1). China accounts for over half of global ESCC cases and deaths annually, underscoring its high burden (2). Despite advancements in traditional treatments such as surgery, radiotherapy, and chemotherapy, the long-term outcomes for ESCC remain suboptimal, with a 5-year overall survival rate of only 20%-30% (3). The emergence of immunotherapy has brought transformative progress to the management of EC, offering new hope for improved outcomes.

The programmed cell death protein 1 (PD-1) receptor, expressed on activated T cells, binds to its ligand programmed cell death ligand 1 (PD-L1), leading to the suppression of T cell proliferation, cytokine secretion, and antitumor immune responses (4). Anti-PD-1 monoclonal antibodies restore T cell-mediated antitumor immunity by inhibiting immune checkpoint pathways. However, due to tumor heterogeneity, PD-L1 expression alone is insufficient as a predictive biomarker for immunotherapy efficacy. For example, the ESCORT-1st study demonstrated that even patients with TPS<1% could benefit from anti-PD-1 therapy combined with chemotherapy (5). These observations underscore the need for in-depth analysis of immune cell subpopulations within the TME to better understand their roles in treatment outcomes.

The TME is a complex ecosystem composed of tumor cells, fibroblasts, immune cells, extracellular matrix, and soluble factors, all of which play critical roles in tumor progression, immune evasion, drug resistance, and metastasis. Immune cells within the TME play dual roles, either suppressing or promoting tumor progression. Among these, CD8^+^ cytotoxic T lymphocytes (CTLs) are critical effectors of antitumor immunity. These cells recognize tumor antigens presented by MHC class I molecules and secrete cytotoxic mediators, such as perforin and granzyme, to eliminate tumor cells (6). However, tumor-infiltrating T lymphocytes (TILs) often display an exhausted phenotype caused by the upregulation of inhibitory receptors such as PD-1 and TIM3 (7). Studies have shown that PD1^+^TIM3^+^ T cells demonstrate impaired proliferation and reduced interferon-γ secretion, but their functionality can be partially restored through the blockade of them (8).

Regulatory T cells (Tregs) also contribute to immune evasion by suppressing effector T cell activity (9). These cells express FOXP3, a transcription factor that regulates their development and immunosuppressive function (10). Research has shown that targeting TIM3 can enhance anti-tumor immune response by reducing Tregs in head and neck cancer (11). Understanding the roles of these molecules is essential for characterizing the immune landscape of ESCC and identifying potential therapeutic targets.

Traditional bulk sequencing methods analyze the average genomic or transcriptomic data from mixed cell populations, obscuring intercellular heterogeneity (12). In contrast, single-cell RNA sequencing (scRNA-seq) enables high-resolution profiling of individual cells, providing detailed insights into immune cell subpopulations and their functional states (13). In this study, scRNA-seq revealed the heterogeneity of the ESCC immune microenvironment and identified distinct immune cell subpopulations with variations in distribution between tumor and adjacent tissues. These findings were further validated through flow cytometry, which highlighted the immune cell subsets most closely associated with the immunotherapy efficacy.

In summary, dynamic monitoring of peripheral blood immune cells using advanced profiling techniques has the potential to enable early prediction of immunotherapy efficacy, providing insights to advance personalized treatment strategies for ESCC.

## Materials and methods

### Patient data and sample collection

Data for this study were retrieved from the Gene Expression Omnibus (GEO) database (https://www.ncbi.nlm.nih.gov/geo/), a public repository maintained by the National Center for Biotechnology Information (NCBI) (14). The raw data were obtained from the GSE145370 dataset, released on October 19, 2020 (15). This dataset includes single-cell RNA sequencing of tumor and adjacent tissues from seven treatment-naïve ESCC patients who underwent surgical resection, providing an unbiased view of the tumor and its microenvironment.

Peripheral blood samples were collected from 20 patients with unresectable locally advanced or metastatic ESCC treated at the First Affiliated Hospital of Soochow University between January 1 and March 31, 2022. All patients received first-line chemotherapy combined with anti-PD-1 monoclonal antibody therapy. Blood samples(3 mL) were drawn from the antecubital vein into EDTA-coated tubes before the first and third treatment cycles. Samples were stored at 4°C and processed within 24 hours. The study was approved by the Ethics Committee of the First Affiliated Hospital of Soochow University, and informed consent was obtained from all participants.

### Single-cell RNA sequencing and analysis

#### Data preparation

The scRNA-seq data from GSE145370 were generated following the protocol by Zheng et al.

#### Data processing

Analysis was performed using the Seurat R package (v3). Quality Control: scRNA-seq has inherent technical limitations, including low transcript coverage and capture efficiency, which can lead to undetectable gene expressions, commonly referred to as “dropouts” (16). Additionally, dead cells may be inadvertently incorporated during library construction, potentially compromising data quality. Cells with a low number of detected genes or a high proportion of mitochondrial gene expression are indicative of ruptured membranes or cell death (17). Therefore, quality control is essential prior to analyzing the raw count matrix to ensure the reliability of the data. Cells with fewer than 300 detected genes or >20% mitochondrial gene content were excluded. Normalization: The NormalizeData function was used to standardize the quality-controlled data. The original count matrix exhibits high dispersion and significant differences in gene expression abundance, necessitating this step to enable meaningful comparisons of gene expression levels across cells. By default, the library size is scaled to 10,000, and values are log-transformed. Specifically, normalized gene expression is calculated as:log1p (10,000 × gene counts/total cell counts). Dimensionality Reduction, Clustering, and Grouping: The FindVariableFeatures function was used to identify highly variable genes (HVGs) for initial dimensionality reduction. The ScaleData function was then applied for data centering, converting standardized expression levels into z-scores to transform the expression matrix into a normal distribution, facilitating subsequent principal component analysis (PCA). Dimensionality reduction was conducted using the RunPCA function, while cell classification was performed using FindNeighbors and FindClusters. To visualize cell subpopulations, the RunUMAP function was applied, and clustering plots based on tissue and patient data were generated using the DimPlot function. [UMAP, Uniform Manifold Approximation and Projection, widely used algorithm for single-cell data analysis and characterized by its high computational efficiency and low memory requirements (18)].

#### Cell annotation

Subpopulations were annotated based on canonical markers:

NK Cells: GNLY, KLRD1

CD4^+^ T Cells: CD3G, CD3D, CD4

CD8^+^ T Cells: CD3G, CD3D, CD8A

B Cells: MS4A1, CD79A, IGLL5, SDC1

Mast Cells: TPSB2, CPA3, TPSAB1

Myeloid DCs: LYZ, APOE

Plasmacytoid DCs: TCF4, IL3RA, PTGDS

Monocytes: LAMP3, PPA1, CST3

Epithelial Cells: KRT19, IFI27, KRT8

#### Differential expression analysis

Dot plots were generated with average expression mapped to color, percent expression mapped to point size, and expression levels represented by Z-score normalized log2(count^+^1) values. Violin plots were also created, with expression levels represented by log2(count^+^1).

The target genes and their corresponding proteins analyzed in this study are presented in **Table 1**. We analyzed CD4 and CD8A (CD8) expression to compare CD4^+^ and CD8^+^ T cells between tumor and adjacent tissues. For CD4^+^ T cells, we assessed TNFRSF4 (CD134), TNFRSF9 (CD137), CD44, SELL (CD62L), and FOXP3 to compare CD134^+^, CD137^+^, CD44^+^, CD62L^+^, and Foxp3^+^ subsets. Similarly, for CD8^+^ T cells, we examined ENTPD1 (CD39), HAVCR2 (TIM3), PDCD1 (PD1), and other markers to compare CD39^+^, TIM3^+^, PD1^+^, CD44^+^, CD62L^+^, CD40L^+^, CD137^+^, and CD103^+^ subsets. Additionally, ITGAE and HAVCR2 expression on dendritic cells (DCs) was evaluated to compare CD103^+^ DCs and TIM3^+^ DCs.

**Table 1:** Target genes and their corresponding proteins.

Gene name	CD8A	TNFRSF4	TNFRSF9	SELL	ENTPD1	HAVCR2	PDCD1	CD40LG	ITGAE
Protein	CD8	CD134	CD137	CD62L	CD39	TIM3	PD1	CD40L	CD103

### Flow cytometry analysis

#### Sample processing

Seven 1.5 mL centrifuge tubes were labeled as Blank, 1, 2, 3, 4, 5, and 6, with the Blank serving as the control. The blank control was included to distinguish between cell autofluorescence and specific fluorescence signals, thereby minimizing false positives.

#### Red blood cell lysis

To each tube, 100 μL of whole blood and 400 μL of red blood cell lysis buffer were added. Samples were mixed thoroughly and incubated at room temperature for 10 minutes to achieve complete lysis. The buffer, primarily consisting of low-osmotic NH_4_Cl, selectively lyses red blood cells while preserving white blood cells.

#### Wash and centrifuge

Cells were washed twice with 900 μL of physiological saline buffer, followed by centrifugation at 500×g for 4 minutes at room temperature. The supernatant was discarded after each wash, leaving a white cell pellet containing leukocytes, including peripheral blood lymphocytes.

#### Antibody staining

Each tube was resuspended in 80 μL of physiological saline. Fluorescein-conjugated antibodies (0.8 μL per tube) were added to all tubes except the Blank control, which received no antibodies. Samples were incubated at 4°C in the dark for 30 minutes. Surface antigen analysis was employed for simplicity, excluding intracellular markers such as Foxp3 due to the additional complexity of membrane permeabilization. The staining scheme was as follows: Blank tube: No antibody, serving as a control. Tube 1: CD8/CD39; Tube 2: CD8/Tim3/PD1/CD40L; Tube 3: CD8/CD44/CD62L; Tube 4: CD3/CD8/CD137; Tube 5: CD4/CD134; Tube 6: CD11c/Tim-3.

This staining setup enables the analysis of the following cell populations: CD39^+^ CD8^+^ T cells, TIM3^+^ CD8^+^ T cells, PD1^+^ CD8^+^ T cells, CD40L^+^ CD8^+^ T cells, CD44^+^ CD62L^+^ CD8^+^ T cells, CD8^+^ CD137^+^ T cells, CD134^+^ CD4^+^ T cells, and TIM3^+^ dendritic cells (DCs).

#### Final centrifugation

After staining, 500 μL of saline was added to each tube, centrifuged at 500×g for 4 minutes, and the supernatant discarded. The pellet was resuspended in 600 μL of saline for flow cytometry analysis.

#### Data acquisition

Flow cytometry analysis was conducted with spectral overlap compensation applied using single-stain controls. Subpopulations, such as TIM3^+^ CD8^+^ T cells, were gated using dual-parameter plots. Fluorescence compensation was performed to address spectral overlap caused by broad fluorophore emissions, ensuring accurate signal detection.

#### Instrument setup

Samples (Blank and tubes 1–6) were processed sequentially using flow cytometry software, with data saved as FCS files. After sample analysis, the instrument was cleaned and shut down according to standard protocols.

### Gating strategy

Flow cytometry data were analyzed using FlowJo software (v10.6.2). Lymphocyte and monocyte populations were initially gated based on forward scatter (FSC) and side scatter (SSC) characteristics to exclude debris. Singlet discrimination was performed using FSC-A versus FSC-H plots to eliminate doublets. CD3^+^ T cells were first identified from the lymphocyte gate, and further divided into CD3^+^CD4^+^ and CD3^+^CD8^+^ subsets. Corresponding markers were analyzed within CD8^+^ and CD4^+^ T cell gates, respectively. The complete gating hierarchy and representative plots are provided in **Supplementary Figure S1**.

### Clinical follow-up

Patients underwent computed tomography (CT) scans every six weeks to evaluate treatment response based on RECIST criteria (19). PFS was defined as the time from the first cycle of anti-PD-1 therapy to disease progression or death. Follow-up continued until January 1, 2023.

### Statistical analysis

Changes in immune cell subsets were calculated as the difference between values obtained from the second and first flow cytometry measurements (denoted as Δ; e.g., Δ_TIM3^+^CD8^+^_). All subset frequencies were expressed as percentages relative to their respective parent populations (e.g., CD3^+^CD8^+^ or CD3^+^CD4^+^ T cells). Patients were stratified by median Δ values to ensure balanced sample sizes for robust comparisons. Kaplan-Meier curves were generated using GraphPad Prism 8, and survival outcomes were assessed using the log-rank test (P < 0.05). Differences in Δ values across clinical response groups (CR^+^PR, SD, and PD) were evaluated using the Kruskal-Wallis test, providing insights into the relationship between immune dynamics and therapeutic response.

## Results

### Data quality control, UMAP visualization, and gene expression

The raw dataset comprised 115,157 cells, of which 110,748 high-quality cells were retained after filtering for low-quality cells. The number of cells per sample ranged from 3,359 to 15,472 per sample, with a median of 1,072 genes detected per cell.

**Figure 1:**
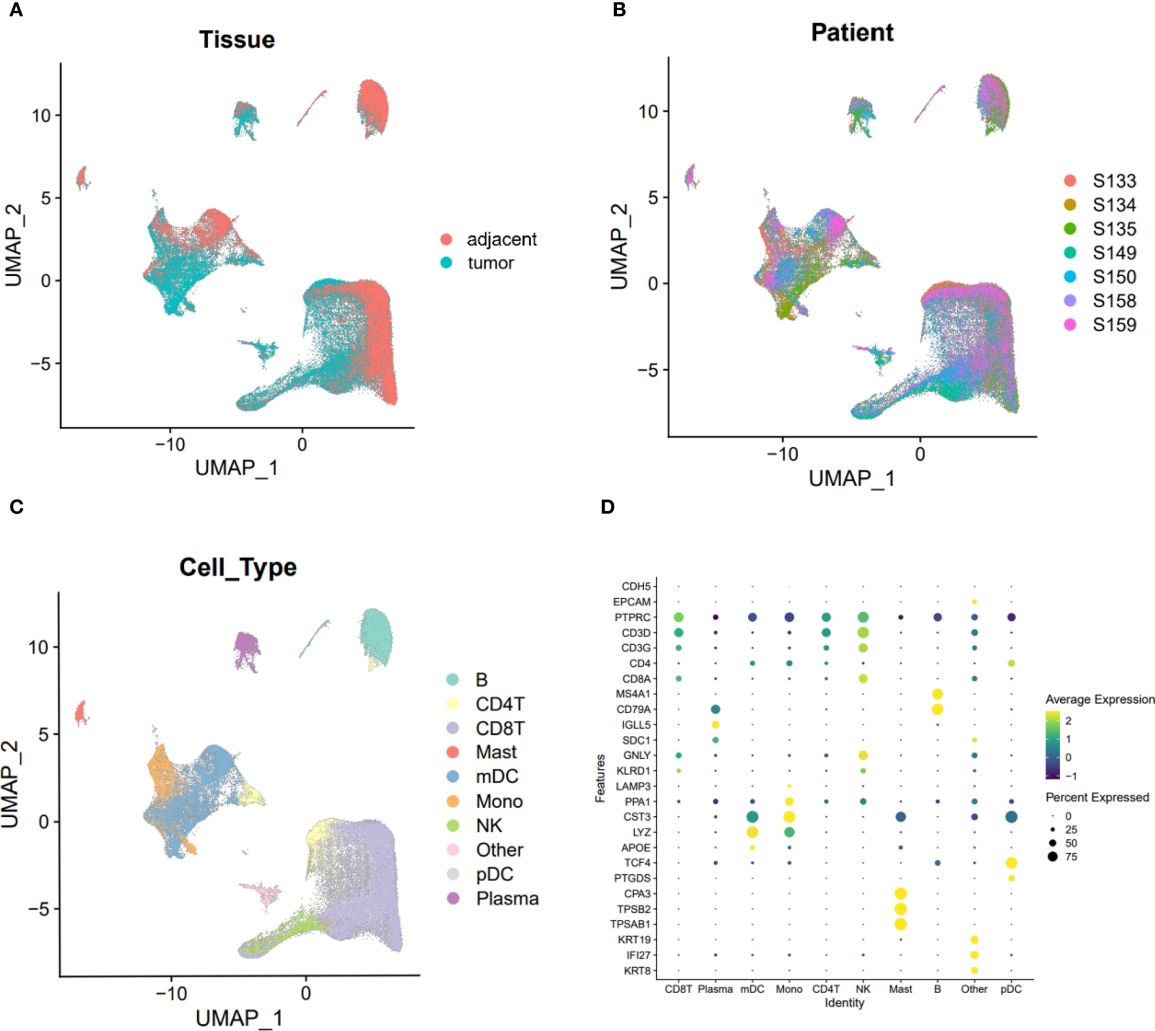
UMAP visualization of cell clusters and gene expression levels across different clusters. **(A)** UMAP plot visualizing cells from tumor and adjacent normal tissues, with distinct clusters representing tissue types. **(B)** UMAP plot visualizing cells from the seven specimens, with different colors indicating the origin of each sample. **(C)** UMAP plot visualizing individual cell clusters, with distinct colors representing different cell populations. **(D)** Dot plot illustrating the expression levels of marker genes across various immune cell subpopulations. Average expression is represented by color intensity, with deeper yellow indicating higher expression levels, while percent expression is mapped to the size of the dots, with larger diameters indicating higher expression percentages.

UMAP plots were employed to visualize cell distribution and clustering. Tissue origins were distinguished by color, with cells from adjacent and tumor tissues displayed in separate colors (**Figure 1A**). Similarly, cells from the seven individual samples were color-coded to indicate their respective sample origins (**Figure 1B**). Subpopulations of cells were identified using marker gene expression, with distinct colors representing each subpopulation (**Figure 1C**). Dot plots further illustrated the relative expression levels of genes across different cell subpopulations, highlighting variations in gene expression profiles (**Figure 1D**).

### Immune remodeling in the tumor microenvironment is characterized by elevated CD8^+^ T cell infiltration

**Figure 2:**
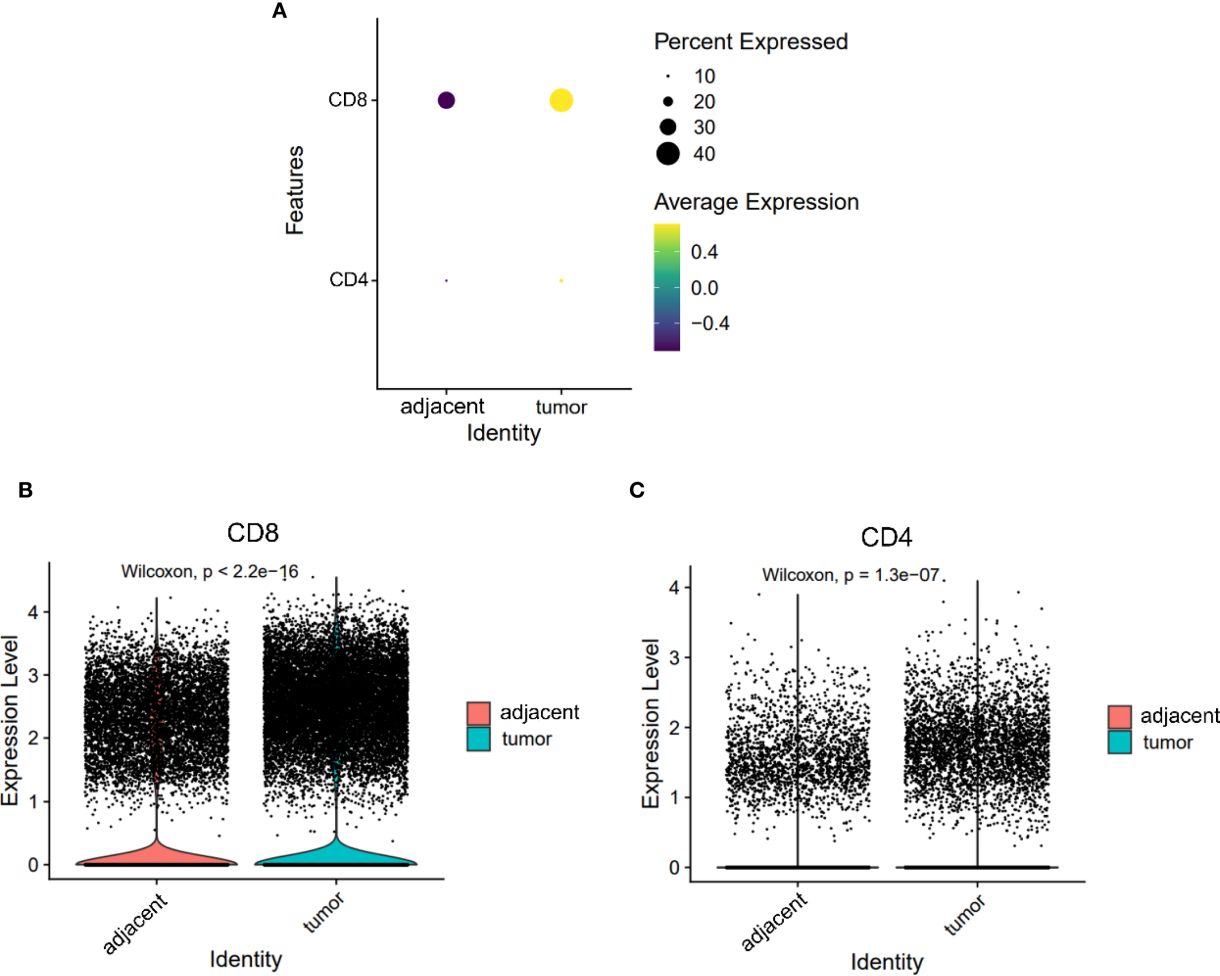
Expression levels of CD4 and CD8 on T cells from adjacent and tumor tissues. **(A)** Overall expression levels of CD4 and CD8 on T cells from adjacent tissues and tumor tissues. **(B, C)** Violin plots depicting the expression differences of CD8 **(B)** and CD4 **(C)** on T cells.

Across both tumor and adjacent tissues, CD8^+^ T cells consistently exhibited higher expression levels than CD4^+^ T cells (**Figure 2A**). Notably, the expression of both CD4 and CD8 was significantly elevated in tumor tissues compared to adjacent tissues (CD8: 1.030 vs. 0.722; CD4: 0.189 vs. 0.139; both P < 0.05, **Figures 2B, C**). These findings indicate a global enrichment of T cells within the TME, with CD8^+^ T cells constituting the predominant subset.

### CD8^+^ T cells in tumors exhibit a distinct exhaustion phenotype

**Figure 3:**
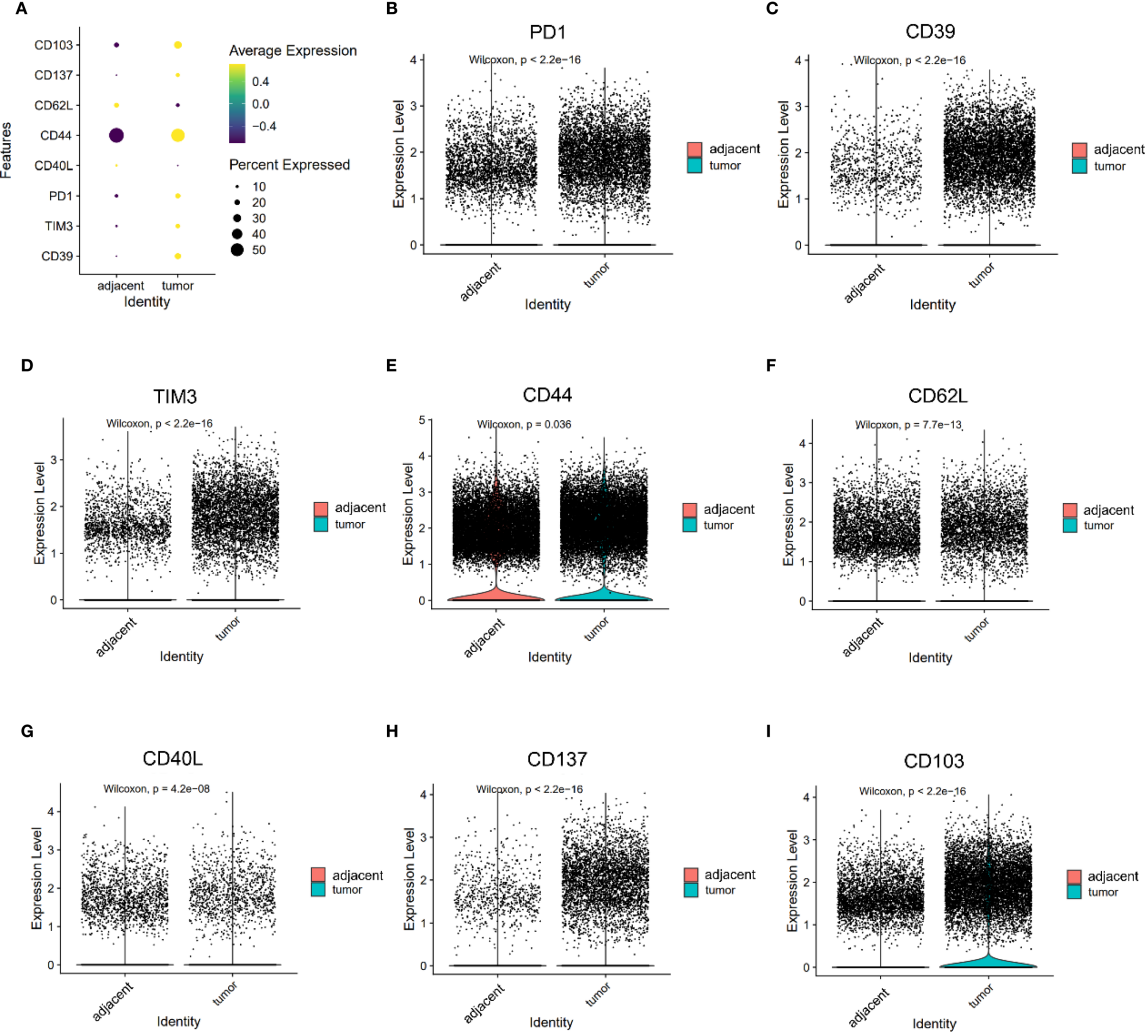
Expression of PD1, CD39, TIM3, CD44, CD62L, CD40L, CD137, and CD103 on CD8^+^ T cells. **(A)** Dot plots illustrating the expression of PD1, CD39, TIM3, CD44, CD62L, CD40L, CD137, and CD103 on CD8^+^ T cells from adjacent and tumor tissues. **(B–F)** Violin plot depicting the expression differences of PD1 **(B)**, CD39 **(C)**, TIM3 **(D)**, CD44 **(E)**, CD62L **(F)**, CD40L **(G)**, CD137 **(H)** and CD103 **(I)** on CD8^+^ T cells between adjacent and tumor tissues.

Tumor-infiltrating CD8^+^ T cells demonstrated significantly higher expression of exhaustion markers compared to their counterparts in adjacent tissues (**Figure 3A**). Specifically, the levels of PD1, CD39, and TIM3 were markedly increased in tumors (PD1: 0.340 vs. 0.203; CD39: 0.431 vs. 0.069; TIM3: 0.292 vs. 0.123; all P < 0.05, **Figures 3B–D**). These changes reflect a shift toward a dysfunctional, exhausted phenotype among cytotoxic T cells within the TME, likely driven by chronic antigen stimulation and immunosuppressive signaling.

### Reduced central memory–like phenotype in tumor-infiltrating T cells

**Figure 4:**
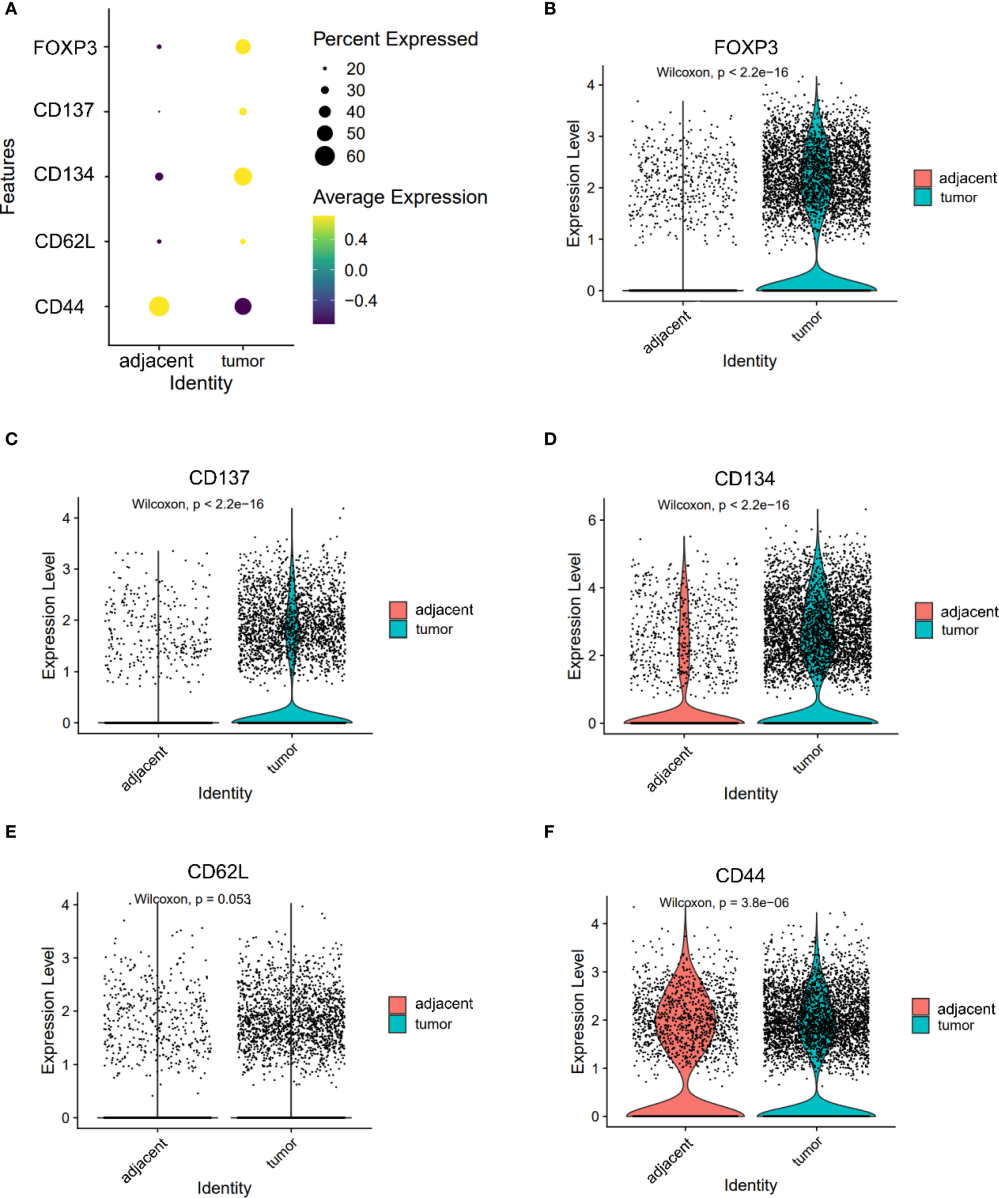
Expression of FOXP3, CD137, CD134, CD62L and CD44 in CD4^+^ T cells. **(A)** Dot plot illustrating the expression of FOXP3, CD137, CD134, CD62L and CD44 in CD4^+^ T cells from adjacent and tumor tissues. **(B–F)** Violin plots depicting the expression differences of FOXP3 **(B)**, CD137 **(C)**, CD134 **(D)**, CD62L **(E)** and CD44 **(F)** in CD4^+^ T cells between adjacent and tumor tissues.

Markers associated with central memory T cells—CD44 and CD62L—were significantly downregulated in tumor-infiltrating CD8^+^ T cells (CD44: 1.150 vs. 1.103, P = 0.036; CD62L: 0.313 vs. 0.250, P < 0.05, **Figures 3E, F**). A similar trend was observed in CD4^+^ T cells, where CD44 expression was significantly reduced in tumors (1.085 vs. 1.242, P < 0.05), while SELL (CD62L) expression trended higher but did not reach statistical significance (0.441 vs. 0.398, P = 0.053, **Figures 4E, F**). CD103 expression on CD8⁺ T cells was significantly elevated in tumor tissues compared with adjacent tissues (P < 0.05, **Figure 3I**). These findings suggest that the TME may impair the maintenance or recruitment of memory T cell subsets, potentially compromising long-term immune surveillance.

### T cell activation markers show divergent expression patterns in CD4^+^ and CD8^+^ compartments

Co-stimulatory receptors exhibited distinct expression profiles between CD4^+^ and CD8^+^ T cells. In the CD4^+^ compartment, both CD134 (1.590 vs. 0.811) and CD137 (0.570 vs. 0.273) were significantly upregulated in tumor tissues (both P < 0.05, **Figures 4C, D**), indicating enhanced activation potential or a regulatory phenotype skew. In contrast, while CD137 was also elevated in tumor-infiltrating CD8^+^ T cells (0.278 vs. 0.055, P < 0.05, **Figure 3H**), CD40L—a key effector molecule facilitating T cell–APC interactions—was significantly reduced in this subset (0.072 vs. 0.129, P < 0.05, **Figure 3G**). These results suggest a partial activation profile in CD8^+^ T cells, potentially limited by suppressed CD40L signaling.

### Regulatory T cells are enriched in the tumor microenvironment

The expression patterns of key immune markers in CD4⁺ T cells were illustrated as a dot plot (**Figure 4A**). FOXP3, a canonical marker of regulatory T cells (Tregs), was significantly upregulated in CD4^+^ T cells from tumor tissues compared to adjacent controls (1.100 vs. 0.469, P < 0.05, **Figure 4B**), indicating an accumulation of immunosuppressive Tregs within the TME.

### Dendritic cell subsets in tumors are skewed toward a dysfunctional phenotype

**Figure 5:**
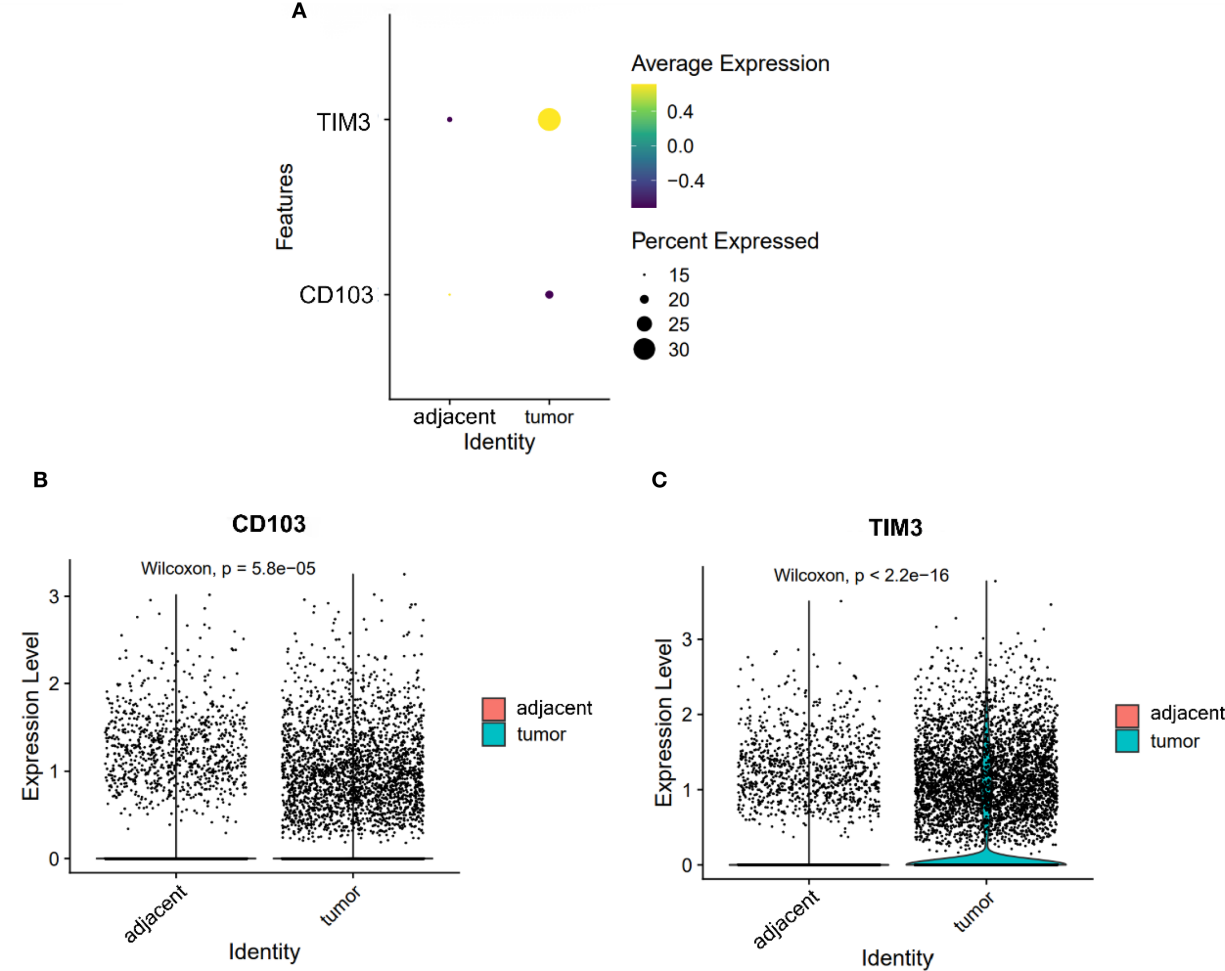
Expression of CD103 and TIM3 on DCs. **(A)** Dot plot illustrating the expression of CD103 and TIM3 on DCs from adjacent and tumor tissues. **(B, C)** Violin plots depicting the expression differences of CD103 **(B)** and TIM3 **(C)** on DCs between adjacent and tumor tissues.

The expression of TIM3 and CD103 on DCs was significantly increased in tumor compared to adjacent tissues (TIM3: 0.368 vs. 0.221; CD103: 0.200 vs. 0.187; both P < 0.05, **Figures 5B, C**). Notably, TIM3 expression exceeded that of CD103 in tumors (**Figure 5A**), suggesting preferential expansion of tolerogenic or dysfunctional DC subsets. This altered DC phenotype may contribute to impaired antigen presentation and the maintenance of T cell exhaustion within the ESCC microenvironment.

### Baseline characteristics and immune cell dynamics in ESCC patients receiving anti-PD-1 therapy

This study included 20 patients with ESCC, with a median age of 60.5 years (range: 44–72; 16 males, 4 females). As of January 1, 2023, 17 patients had reached the study endpoint, with a median progression-free survival (mPFS) of 6.15 months. None of the patients achieved a complete response (CR). Partial responses (PR) were observed in 7 patients, stable disease (SD) in 10 patients, and progressive disease (PD) in 3 patients. The clinical characteristics of ESCC patients are detailed in **Supplementary Table S1**. The dynamic changes in peripheral immune cell subpopulations following immunotherapy in ESCC patients are presented in **Supplementary Table S2**.

### Changes in CD8^+^ T cell subsets and their association with PFS and treatment efficacy

#### Exhausted CD8^+^ T cells: TIM3^+^, PD-1^+^, and CD39^+^ subsets

**Figure 6:**
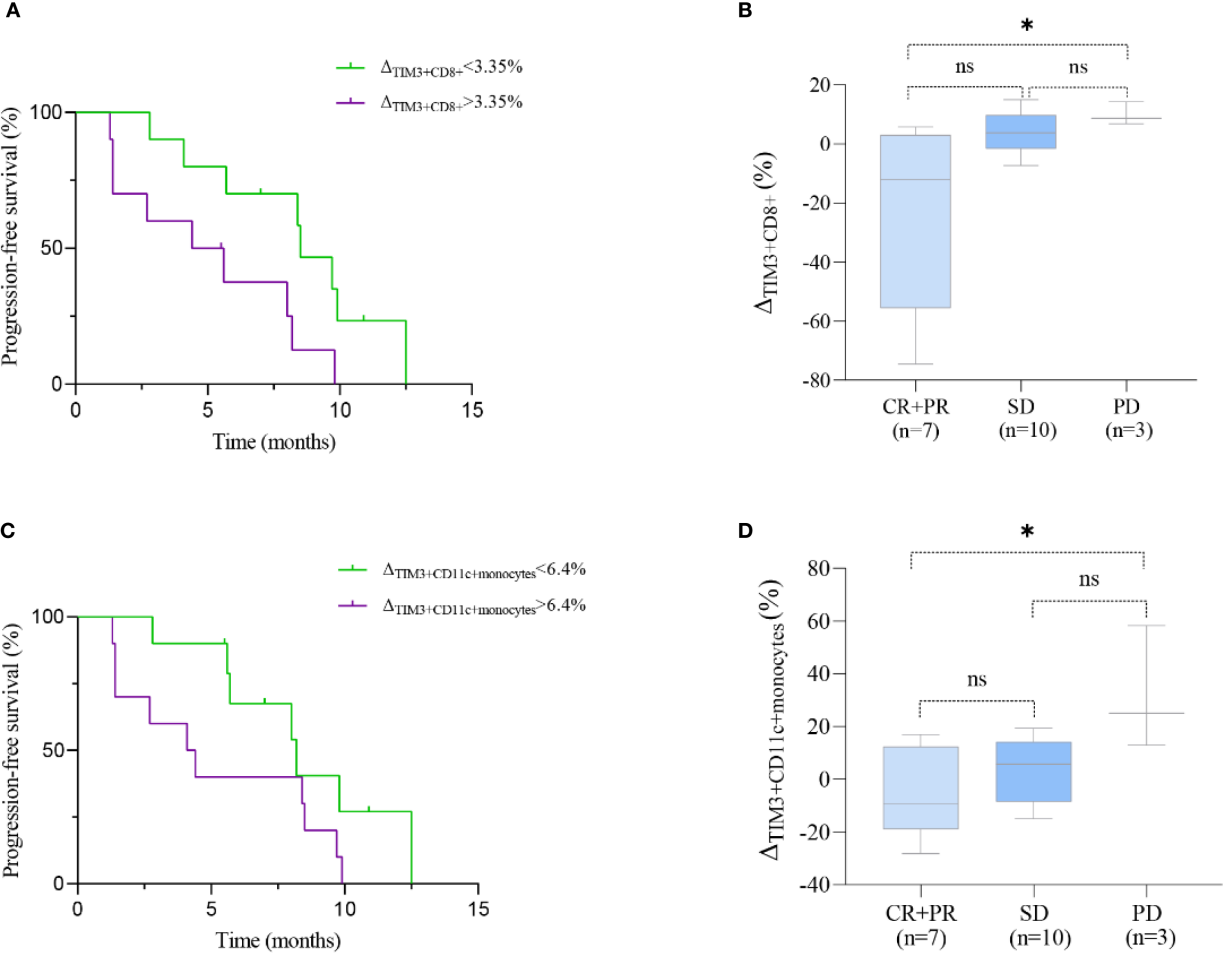
Prognostic significance of TIM3^+^ immune subsets in ESCC patients treated with immunotherapy. **(A, C)** Kaplan–Meier curves showing progression-free survival (PFS) stratified by median changes (Δ) in TIM3^+^CD8^+^ T cells **(A)** and TIM3^+^CD11c^+^ monocytes **(C)**. **(B, D)** Boxplots comparing the change (Δ) in these subsets across clinical response groups: CR/PR (complete/partial response), SD (stable disease), and PD (progressive disease). Statistical comparisons were performed using the log-rank test (PFS) and Kruskal–Wallis test with Dunn’s *post hoc* test (boxplots). *P < 0.05; ns, not significant.

**Figure 7:**
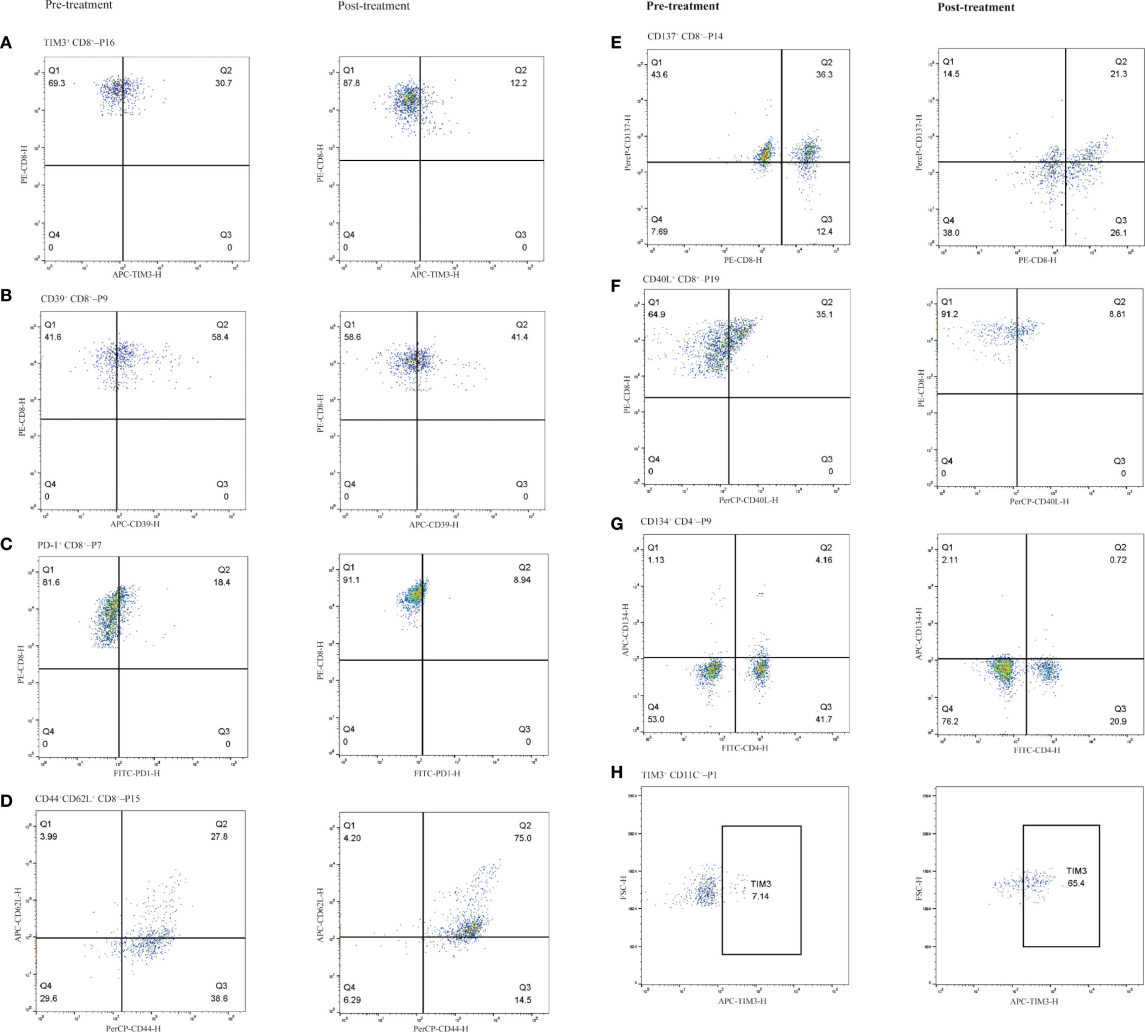
Representative immune cell subpopulations before and after immunotherapy in selected patients, grouped by cell type. CD8^+^ T cell subsets **(A–F)**: **(A)** TIM-3^+^ CD8^+^ T cells (Patient 16), **(B)** CD39^+^ CD8^+^ T cells (Patient 9), **(C)** PD-1^+^ CD8^+^ T cells (Patient 7), **(D)** CD44^+^CD62L^+^ CD8^+^ T cells (central memory phenotype; Patient 15), **(E)** CD137^+^ CD8^+^ T cells (Patient 14), **(F)** CD40L^+^ CD8^+^ T cells (Patient 19). CD4^+^ T cell subset **(G)** CD134^+^ CD4^+^ T cells (Patient 9). CD11C^+^ monocyte subset **(H)**: **(H)** TIM-3^+^ dendritic cells (Patient 1). Left column shows pre-treatment samples; right column shows post-treatment samples.

In patients treated with immunotherapy, the median change (Δ) in TIM3^+^ CD8^+^ T cell levels was 3.35% (range: -74.6% to 15.0%). Patients with a change (Δ) >3.35% experienced shorter PFS compared to those with Δ<3.35% (5.0 vs. 8.5 months; HR = 2.691, 95% CI 0.949–7.633, P = 0.024, **Figure 6A**). **Figure 7A** shows representative flow cytometry data from patient 16.

The median Δ in CD39^+^ CD8^+^ T cell levels was 3.45% (range: -22.9% to 26.6%). Patients with Δ>3.45% showed a trend toward longer PFS compared to those with Δ<3.45% (8.0 vs. 7.0 months; HR = 0.637, 95% CI 0.243–1.671, P = 0.318, **Supplementary Figure S3a**). **Figure 7B** shows representative flow cytometry data from patient 9.

The median Δ in PD1^+^ CD8^+^ T cell levels was 0.6% (range: -9.5% to 23.3%). Patients with Δ>0.6% tended to have shorter PFS compared to those with Δ<0.6%, though the difference was not statistically significant (5.6 vs. 8.5 months; HR = 1.722, 95% CI 0.631–4.694, P = 0.229; **Supplementary Figure S3c**). **Figure 7C** shows representative flow cytometry data from patient 7.

The Kruskal-Wallis test revealed significant differences in ΔTIM3^+^ CD8^+^ levels among therapeutic response groups (P = 0.004). Specifically, patients achieving CR or PR exhibited significantly lower ΔTIM3^+^ CD8^+^ levels compared to those with PD (P = 0.018). However, differences between CR^+^PR and SD groups, as well as between SD and PD groups, were not statistically significant (P = 0.061, 0.754, respectively; **Figure 6B**).No significant differences in ΔCD39^+^ CD8^+^ levels and ΔPD1^+^ CD8^+^ levels were noted among therapeutic response groups (**Supplementary Figures S3b, d**).

### Activated CD8^+^ T cells: CD137^+^ and CD40L^+^ subsets

The median Δin CD137^+^ CD8^+^ T cell levels was 2.7% (range: -15.0% to 11.2%). Patients with Δ>2.7% had a trend toward longer PFS compared to those with Δ<2.7%, but the difference was not statistically significant (8.2 vs. 6.9 months; HR = 0.624, 95% CI 0.238–1.640, P = 0.782; **Supplementary Figure S3e**). **Figure 7E** shows representative flow cytometry data from patient 14.

The median Δ in CD40L^+^ CD8^+^ T cell levels was -1.35% (range: -26.3% to 6.4%). Patients with Δ>-1.35% tended to have shorter PFS compared to those with Δ<-1.35%, but the difference was not statistically significant (6.9 vs. 8.4 months; HR = 1.902, 95% CI 0.711–5.087, P = 0.154; **Supplementary Figure S3g**). **Figure 7F** shows representative flow cytometry data from patient 19. No significant differences in ΔCD40L^+^ CD8^+^ levels and ΔCD137^+^ CD8^+^ levels were observed among groups with response groups (**Supplementary Figures S3f, h**).

### Central memory CD8^+^ T cells: CD44^+^CD62L^+^ subset

Following immunotherapy, the median Δin CD44^+^ CD62L^+^ CD8^+^ T cell levels was 7.65% (range: -26.7% to 47.2%). Patients with Δ>7.65% showed a trend toward shorter PFS compared to those with Δ <7.65%, though the difference was not statistically significant (5.0 vs. 8.2 months; HR = 1.180, 95% CI 0.456–3.054, P = 0.720; **Supplementary Figure S3k**). **Figure 7D** shows representative flow cytometry data from patient 15. No significant differences in ΔCD44^+^ CD62L^+^ CD8^+^ levels were observed among response groups (**Supplementary Figure S3l**).

### Changes in CD4^+^ T cell subsets and their association with PFS and treatment efficacy

In ESCC patients receiving immunotherapy, the median Δ in CD134^+^CD4^+^ T cell levels was 0.85% (range: -3.4% to 4.3%). Patients with Δ>0.85% showed a trend toward longer PFS compared to those with Δ<0.85%, though the difference was not statistically significant (8.1 vs. 5.7 months; HR = 0.809, 95% CI 0.309–2.120, P = 0.650; **Supplementary Figure S3i**). **Figure 7G** shows representative flow cytometry data from patient 9. No significant differences in ΔCD134^+^CD4^+^ T cell levels were observed among therapeutic response groups (P > 0.05, **Supplementary Figure S3j**).

### Changes in CD11c^+^ monocytes and their association with PFS and treatment efficacy

In ECC patients receiving immunotherapy, the median change Δ in CD11c^+^ monocytes levels was 6.4% (range: -28.2% to 58.3%). Patients with Δ>6.4% exhibited a trend toward shorter PFS compared to those with Δ <6.4%, although the difference was not statistically significant (4.3 vs. 8.2 months; HR = 2.203, 95% CI 0.833–5.829, P = 0.088; **Figure 6C**). **Figure 7H** shows representative flow cytometry data from patient 1. Significant differences in ΔTIM3^+^ CD11c^+^ monocytes levels were observed across groups with different therapeutic responses (P = 0.039). Specifically, patients achieving CR or PR showed significantly lower ΔTIM3^+^ DC levels compared to those with PD (P = 0.042). However, no statistically significant differences were observed between CR^+^PR and SD groups or between SD and PD groups (P > 0.05, **Figure 6D**).

## Discussion

Variability in the response to anti-PD-1 therapy among ESCC patients is closely linked to immune cell heterogeneity within the TME (20). While single-cell sequencing has revealed cellular diversity and trajectories in the TME (21), the functional roles of targets such as TIM3 remain underexplored. This study examines immune marker expression in tumor-infiltrating and adjacent cells, providing new insights into ESCC immunobiology.

Consistent with an active anti-tumor immune response, we observed robust infiltration of CD8^+^ T cells in ESCC tumor tissues. However, these tumor-infiltrating CD8^+^ T cells exhibited high levels of exhaustion markers—PD-1, CD39, and TIM3, compared to adjacent tissues. Chronic antigen stimulation and suppressive cues within the TME can drive TILs toward a dysfunctional state (22, 23), characterized by sustained upregulation of inhibitory receptors such as PD-1, TIM-3, and CD39 (24). Exhausted CD8^+^ T cells produce less IL-2, IFN-γ, and TNF-α and display impaired proliferative capacity (25). This exhaustion diminishes antitumor activity and reflects localized immune dysfunction (26). In a diffuse large B-cell lymphoma model, blockade of PD-1 or TIM-3 restored cytokine production and proliferation of these exhausted CD8^+^ T cells (8). Thus, the prevalence of exhausted TILs in ESCC provides a mechanistic explanation for limited PD-1 monotherapy efficacy and suggests that reversing T cell exhaustion is key to improving outcomes.

ESCC exhibited a marked enrichment of CD8^+^ tissue-resident memory T cells (TRM), characterized by the expression of CD103, a defining integrin that mediates epithelial retention. CD103 binds to E-cadherin expressed on carcinoma cells, thereby promoting the stable localization of TRM within the TME (27). TRM cells are stationed at tumor sites for immediate effector function upon antigen re-encounter and have been associated with improved anti-tumor immunity and response to checkpoint therapy in multiple cancers (28). The abundance of CD103^+^ TRM in ESCC suggests that local immunity remains partially preserved, potentially enabling initial tumor recognition. However, this local residency was accompanied by a marked reduction in CD44^+^CD62L^+^ central memory T cells (Tcm). Tcm reside in secondary lymphoid organs, mediate long-term immune memory, and mount proliferative recall responses upon antigen re-stimulation (29). The diminished Tcm population in ESCC suggests that the T cells in tumors are skewed toward either short-lived effector or terminally exhausted states. This diminished Tcm population in tumors may impair immune system “reservoirs” that normally support durable responses and rapid recall upon tumor antigen recurrence (30).

Adding further complexity, co-stimulatory receptors CD137 (4-1BB) and CD134 (OX40) were upregulated on tumor-infiltrating CD8^+^ and CD4^+^ T cells, respectively. CD137 enhances T cell proliferation and cytokine production upon ligand engagement (31), and agonists targeting 4-1BB are being investigated to boost anti-tumor immunity (32). Notably, OX40 signaling has been shown to destabilize FOXP3^+^ Tregs and attenuate their suppressive capacity (33). Despite the upregulation of OX40, the TME in ESCC remained enriched with FOXP3^+^ Tregs, suggesting persistent immunosuppression (34). The concurrent presence of activated effector T cells and expanded Tregs in ESCC reflects a competitive immunological landscape.

TIM3, encoded by the HAVCR2 gene, is a transmembrane protein initially identified on Th1 and Tc1 cells but also expressed on DCs (35, 36). Binding of Galectin-9 to TIM3 induces the release of BAT3 from the intracellular tail of TIM3, leading to T cell apoptosis (37). TIM3 upregulation on T cells following PD-1 blockade has been observed in various cancers, correlated with tumor recurrence in preclinical models (38). Consistently, our findings show that patients with shorter PFS exhibited greater expansion of TIM3^+^CD8^+^ T cells after immunotherapy, reinforcing the role of TIM3 in immune resistance.

Taking these findings together, we propose a model of immunotherapy resistance in ESCC centered on TIM-3. In the tumor, an abundance of TIM-3^+^ exhausted CD8^+^ TILs create an immune milieu prone to tumor immune escape. Upon PD-1 blockade, patients with a highly suppressive TME mount only transient T-cell reinvigoration, after which TIM-3–mediated pathways blunt the response, leading to adaptive resistance. These patients show peripheral immune changes: increasing frequencies of circulating TIM-3^+^ CD8^+^ T cells that reflect ongoing T-cell dysfunction. Clinically, this translates into poor outcomes. This model integrates local and systemic immune signatures, revealing how their convergence dictates response to PD-1 blockade. It is consistent with prior observations in gastrointestinal cancers that “immune-hot” tumors with reinvigorated T cells respond, whereas “immune-cold/exhausted” tumors evade therapy through alternate checkpoints and suppressive cells (39).

Our results underscore that TIM-3 is not only a biomarker of T-cell exhaustion but also a potentially druggable target for overcoming resistance in ESCC. TIM-3’s role in dampening immune responses has been documented across multiple tumors, and it appears especially pertinent in the context of PD-1 blockade failure (40, 41). Encouragingly, therapeutic targeting of TIM-3 is already underway: sabatolimab, an anti–TIM-3 monoclonal antibody, received FDA Fast Track designation for advanced solid tumors (42), and early-phase trials combining TIM-3 and PD-1 blockade have shown acceptable safety and preliminary efficacy (43). Our finding that TIM-3^+^ cells associate with poorer outcomes provides a strong rationale to test such combination strategies in ESCC. We speculate that dual-checkpoint inhibition (anti–PD-1 plus anti–TIM-3) could reinvigorate exhausted TILs more completely than PD-1 blockade alone, converting partial responders into durable responders.

Despite the insights gained, several limitations should be acknowledged. First, the small, single-center cohort (N = 20) limits statistical power. Second, as an observational study, residual confounding is unavoidable. Key clinical variables (e.g. PD-L1 status) were not fully controlled. Third, although we integrated public tumor scRNA-seq data with prospective blood profiling, the absence of an external validation cohort limits the robustness of the proposed prognostic threshold, which should be considered exploratory. Finally, no functional assays were performed to establish causality. The causal role of TIM-3^+^ T cells in immune resistance remains uncertain, and mechanistic studies are required to determine whether they are active mediators or passive markers.

In summary, our study identifies TIM-3^+^ CD8^+^ T cells as key immunological features linking the TME to systemic immune changes and clinical outcomes. Patients whose tumors foster a TIM-3–high, exhausted immune contexture are more likely to exhibit peripheral T-cell dysfunction during therapy and to experience disease progression, underscoring TIM-3’s role in adaptive resistance to PD-1 inhibitors. From a translational perspective, dynamic monitoring of TIM-3^+^ T cells in blood could serve as an non-invasive indicator of immunotherapy efficacy in ESCC, aiding in treatment decisions. More importantly, our work lays a biological foundation for therapeutically targeting TIM-3 in ESCC and supports its integration into combinatorial immunotherapy to overcome resistance and improve clinical outcomes.

## Data Availability

The single-cell RNA sequencing dataset analyzed during the current study is publicly available in the Gene Expression Omnibus (GEO) database under accession number GSE145370. Clinical and flow cytometry data generated and analyzed during this study are available from the corresponding author upon reasonable request.
