# Supplementary material for: Correlation of immune cell subsets in the tumor microenvironment and peripheral blood with immunotherapy response in esophageal squamous cell carcinoma
Source: Front Immunol. 2025 Oct 17;16:1633748. doi: 10.3389/fimmu.2025.1633748 (PMC12575347; doi:10.3389/fimmu.2025.1633748)
Supplement: Supplementary file 1 [file DataSheet1.docx]

**Correlation of Immune Cell Subsets in the Tumor Microenvironment and Peripheral Blood with Immunotherapy Response in Esophageal Squamous Cell Carcinoma**

Table S1. Clinical Characteristics of Patients with ESCC 2

Table S2. Changes in Peripheral Immune Cell Subpopulations in ESCC Patients (n=20) Following Immunotherapy 3

Figure S1. Gating strategy for flow cytometry analysis 4

Figure S2. Representative dot plots validating marker co-expression and spectral separation………….6

Figure S3. Functional classification of additional immune subsets analyzed for association with progression-free survival (PFS) and clinical response in ESCC patients treated with immunotherapy ...8

**Table S1. Clinical Characteristics of Patients with ESCC**

| **Clinical Characteristic** | **Number of Patients (Percentage)** |
| --- | --- |
| Sex |  |
| Male | 16 (80%) |
| Female | 4 (20%) |
| Age (Years) |  |
| <60 | 9 (45%) |
| ≥60 | 11 (55%) |
| History of Radiation Therapy |  |
| Yes | 7 (35%) |
| No | 13 (65%) |
| History of Curative Surgery |  |
| Yes | 4 (20%) |
| No | 16 (80%) |

**Table S2. Changes in Peripheral Immune Cell Subpopulations in ESCC Patients (n=20) Following Immunotherapy**

| Patient | Response | TIM3^+^CD8^+^ | CD39^+^CD8^+^ | PD1^+^CD8^+^ | CD40L^+^CD8^+^ | CD44^+^CD62L^+^CD8+ | CD137^+^CD8^+^ | CD134^+^CD4^+^ | TIM3^+^CD11c^+^ monocytes |
| --- | --- | --- | --- | --- | --- | --- | --- | --- | --- |
| 1 | PD | 14.3 | 6.5 | 8.5 | 3.9 | 8.9 | 11.1 | -1.2 | 58.3 |
| 2 | PD | 6.8 | -5.5 | -9.2 | -6.5 | 12.5 | 10.8 | 4.3 | 25.0 |
| 3 | PD | 8.6 | 26.6 | -6.5 | 6.3 | 6.8 | 11.2 | -3.4 | 12.9 |
| 4 | SD | 15.0 | -6.7 | 9.2 | 2.6 | -16 | -5.2 | 0.9 | 13.0 |
| 5 | PR | -12.0 | -19.7 | 17.6 | 5.8 | 10.0 | 2.0 | 1.0 | -19.0 |
| 6 | PR | 3.1 | -3.6 | -3.6 | -20.4 | 13.4 | -6.8 | 0.8 | 17.0 |
| 7 | SD | 10.6 | 11.6 | 23.3 | -14.6 | 18.5 | 2.6 | 1.8 | 19.4 |
| 8 | SD | 9.5 | 9.7 | 10.4 | -7.5 | -23.5 | 4.5 | -3.1 | -14.9 |
| 9 | SD | 6.5 | -17.0 | 13.4 | -11.5 | 8.7 | 6.7 | -3.4 | -6.4 |
| 10 | SD | 1.7 | 18.2 | 13.4 | 4.2 | -10.5 | -10.0 | 0.3 | 3.8 |
| 11 | PR | -10.2 | 1.7 | 17.7 | 2.8 | 18.0 | -5.5 | -1.5 | -18.3 |
| 12 | SD | 3.6 | 6.3 | -1.4 | 5.0 | -9.6 | -0.4 | 3.9 | -5.8 |
| 13 | PR | 5.8 | 12.3 | 20.1 | 3.6 | -15.3 | 8.9 | 4.2 | -9.5 |
| 14 | SD | -7.3 | -10.6 | -7.5 | -5.3 | -22.0 | -15.0 | -3.3 | 13.5 |
| 15 | SD | -6.9 | 2.4 | -3.8 | -9.5 | 47.2 | 7.6 | 3.8 | 15.9 |
| 16 | PR | -18.5 | -22.9 | -8.8 | 3.3 | -26.7 | -5.8 | 2.0 | 12.4 |
| 17 | SD | 3.9 | -8.4 | -5.3 | 6.4 | -9.2 | -3.8 | -0.6 | -15.0 |
| 18 | SD | 0.3 | 4.5 | 2.6 | -19.6 | 8.5 | 8.4 | -2.8 | 7.8 |
| 19 | PR | -55.6 | 9.8 | -9.5 | -26.3 | -20.5 | 10.0 | 2.5 | 5.0 |
| 20 | PR | -74.6 | 5.2 | -1.9 | -15.7 | 11.8 | 2.8 | 1.5 | -28.2 |

Values represent the percentage change (Δ%) in the frequency of each marker-positive immune subset between pre-treatment and post-treatment peripheral blood samples. For each marker, changes were calculated within the corresponding parent population: CD8⁺ T cells (TIM3⁺, CD39⁺, PD1⁺, CD40L⁺, CD44⁺CD62L⁺, CD137⁺), CD4⁺ T cells (CD134⁺), and CD11c⁺ monocytes (TIM3⁺ CD11c⁺ monocytes). PR, Partial Response. PD, Progressive Disease. SD, Stable Disease.


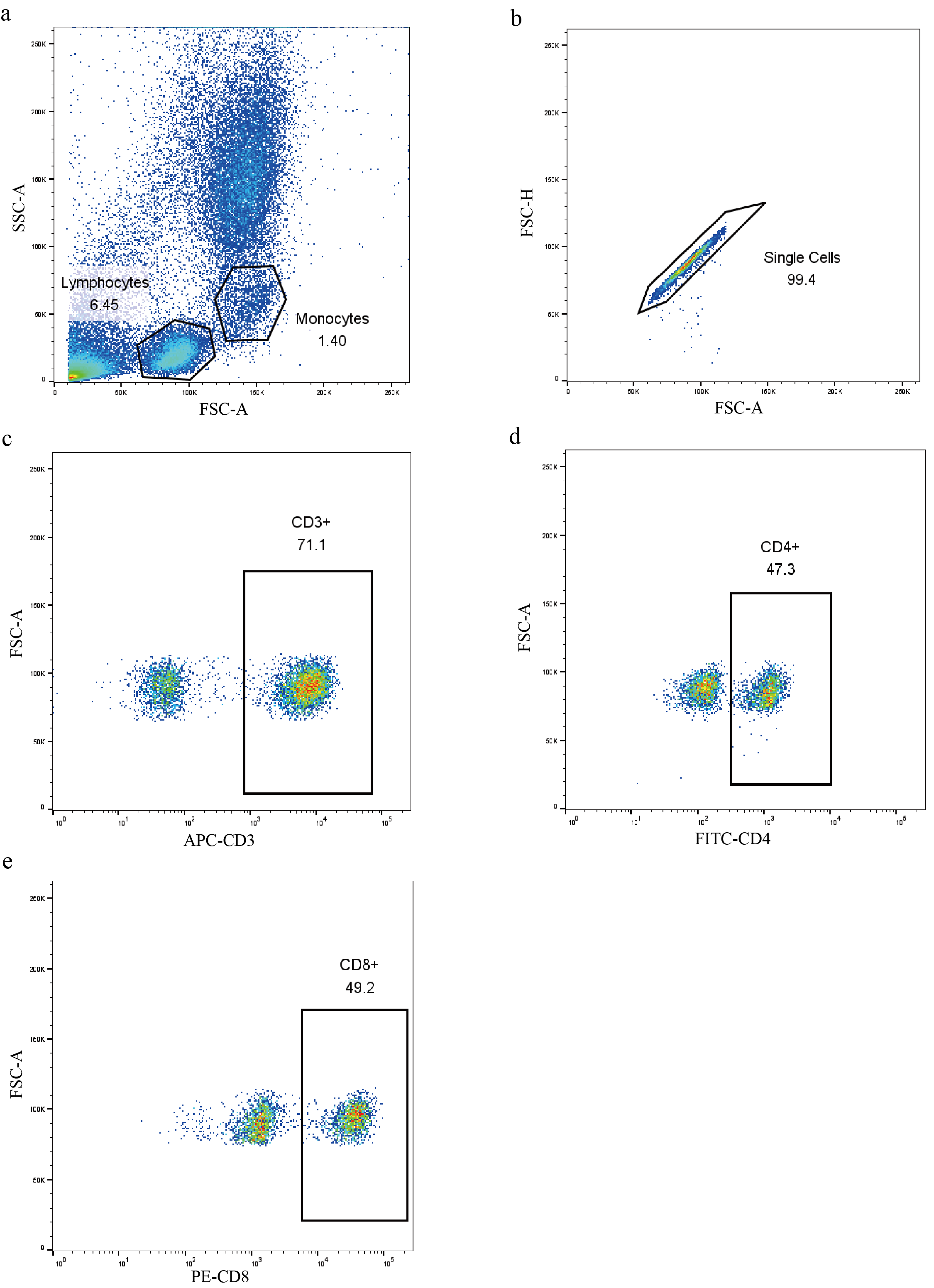


Figure S1. Gating strategy for flow cytometry analysis

(a) Forward scatter area (FSC-A) versus side scatter area (SSC-A) plot to identify lymphocytes and monocytes based on cell size and granularity.

(b) Singlet cells were selected by plotting FSC-A versus FSC-H to exclude doublets.

(c) CD3⁺ T cells were gated from the singlet lymphocyte population.

(d) CD4⁺ T cells were identified within the CD3⁺ population based on FITC-conjugated CD4 expression.

(e) CD8⁺ T cells were identified within the CD3⁺ population based on PE-conjugated CD8 expression.

Figure S2. Representative dot plots validating marker co-expression and spectral separation.

Dot plots show co-expression of PD1 (FITC), TIM3 (APC), and CD40L (PerCP) with CD8 (PE) in CD8⁺ T cells. Left panels (a, c, e) represent blank (unstained) control tubes used to determine background fluorescence and set gating thresholds. Right panels (b, d, f) display corresponding stained samples, demonstrating clear resolution of double-positive (Q2) populations without evidence of signal spillover or spectral overlap. Gates were drawn based on the negative tail of blank controls and consistently applied across samples.

Figure S3. Functional classification of additional immune subsets analyzed for association with progression-free survival (PFS) and clinical response in ESCC patients treated with immunotherapy.

A: Exhausted T cells: Kaplan–Meier survival curves and boxplots for CD39⁺CD8⁺ T cells (S1a, S1b) and PD1⁺CD8⁺ T cells (S1c, S1d).

B: Activated T cells: CD137⁺CD8⁺ (e, f), CD134⁺CD4⁺ (g, h), and CD40L⁺CD8⁺ T cells (i, j).

C: Central memory CD8⁺ T cells defined by CD44⁺CD62L⁺ expression (k, l).

Kaplan–Meier plots show PFS stratified by whether the change in each immune subset (Δ) was above or below the median cutoff.

Boxplots compare Δ values across clinical response groups: CR/PR (complete/partial response), SD (stable disease), and PD (progressive disease).

Statistical analyses were performed using the log-rank test (Kaplan–Meier) and Kruskal–Wallis test with Dunn’s post hoc test (boxplots).

All comparisons were not statistically significant (*P > 0.05; ns = not significant).
